# Experimental Animal Models for Moyamoya Disease: A Species-Oriented Scoping Review

**DOI:** 10.3389/fsurg.2022.929871

**Published:** 2022-07-01

**Authors:** Lei Cao, Yang Dong, Kaiwen Sun, Dongpeng Li, Hao Wang, Hongwei Li, Bo Yang

**Affiliations:** Department of Neurosurgery, The First Affiliated Hospital of Zhengzhou University, Zhengzhou, China

**Keywords:** moyamoya disease, animal models, genetic approach, surgical approach, immunological/inflammatory approach, review

## Abstract

Moyamoya disease (MMD) is a rare cerebrovascular disease characterized by progressive stenosis of large intracranial arteries and a hazy network of basal collaterals called moyamoya vessels. The etiology and pathogenesis of MMD are still obscure. The biggest obstacles in the basic research of MMD are difficulty in obtaining specimens and the lack of an animal model. It is necessary to use appropriate and rationally designed animal models for the correct evaluation. Several animal models and methods have been developed to produce an effective MMD model, such as zebrafish, mice and rats, rabbits, primates, felines, canines, and peripheral blood cells, each with advantages and disadvantages. There are three mechanisms for developing animal models, including genetic, immunological/inflammatory, and ischemic animal models. This review aims to analyze the characteristics of currently available models, providing an overview of the animal models framework and the convenience of selecting model types for MMD research. It will be a great benefit to identify strategies for future model generations.

## Introduction

Moyamoya disease (MMD) is a chronic cerebrovascular disease characterized by progressive occlusion of the terminal internal carotid artery (ICA) and the secondary formation of collateral vessels. Suzuki and Takaku first described this rare vascular disease (1). The term “moyamoya” refers to the unusual vascular collaterals at the base of the brain. Cerebral angiography is the gold standard investigation of MMD for diagnosis contrast to the tumors. MMD is found worldwide; China, Korea, and Japan have the highest incidence around the world (2). To date, the detailed underlying etiology and pathogenesis mechanisms are still poorly understood. Except for progressive narrowing of the ICA and circle of Willis, cortical microvascularization is a specific finding; increased microvascular density and diameter were observed in patients. However, neither the nature of moyamoya vessels, “newly formed” perforator arteries, nor the remodeling and dilatation of pre-existing vascular channels has not been determined. The histopathological changes in the distal ICA reported hyperplastic smooth muscle cells or endothelium and stenotic or occlusive lesions related to the fibrocellular thickening of the intima (3); pathological findings include fibrin deposition, elastic layer fragments, and media weakening. It is a fact that the difficulty in obtaining specimens and the lack of an animal model make it difficult to carry out the mechanism research of MMD.

Currently, much attention has been paid to the animal models for MMD. However, few articles focus on the current situation. Using appropriate and rational animal models is necessary for the correct evaluation. Numerous animal species and methods have been developed to produce an effective MMD model, each with its advantages and disadvantages. We will discuss the current state of experimental animal models for MMD in this review. As far as we know, this is the first attempt to categorize MMD models based on the animal species.

## Methods

### Protocol and Registration

The umbrella review was based on the internationally accepted Preferred Reporting Items for Systematic Reviews and Meta-Analyses guidelines. The review protocol was not registered.

### Sources and Search

In January 2022, a literature search was performed on PubMed, Scopus, Web of Science, and Google Scholar databases. The following search terms with various combinations were used: “moyamoya,” “animal,” “model,” “zebrafish,” “rat,” “mouse,” “cat,” “rabbit,” “monkey,” “dog,” “pig,” and “cell.”

### Eligibility Criteria

Articles were included for analysis of animal models to explicitly address MMD pathogenesis and/or to evaluate experimental treatments relevant to MMD. Articles were excluded if the animal model did not explicitly assess the cerebrovasculature.

### Selection of Evidence and Synthesis of Results

**Figure 1:**
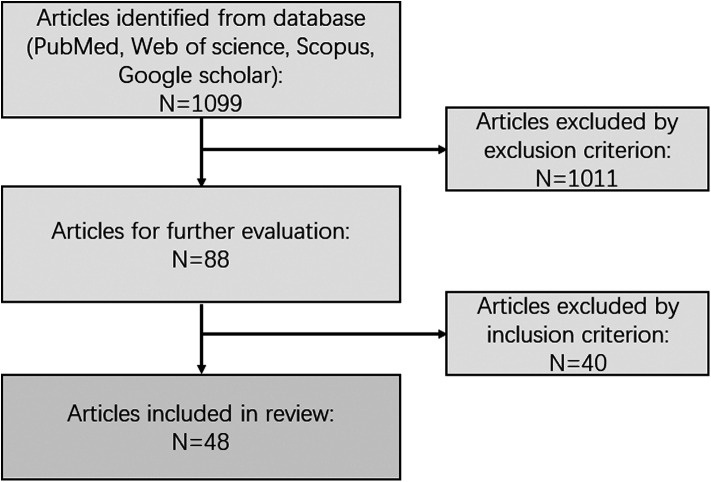
Flowchart schematic of papers identified by the search criteria.

Two reviewers screened independently, and duplicates were removed manually. Results were grouped based on species. This review’s dominating points cover data regarding animal model species, techniques, assessment methods, relevant histopathology, and outcomes (Figure 1).

## Results

**Figure 2:**
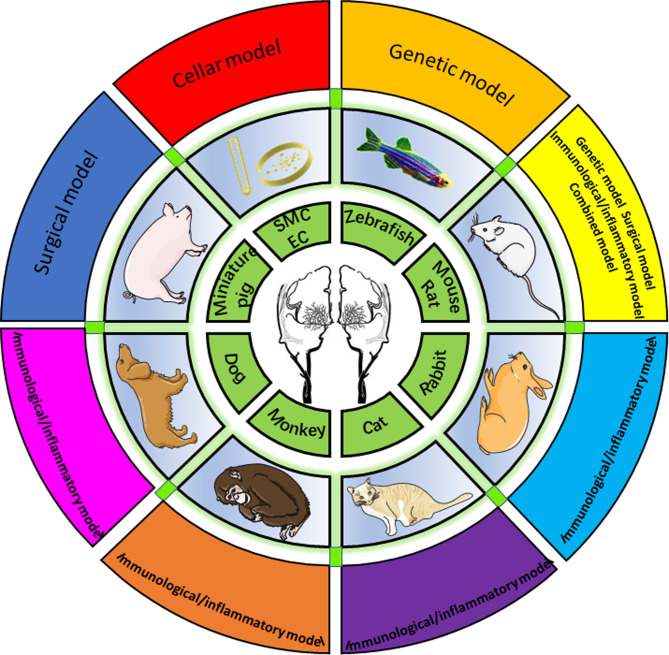
Eight animal models summarized by species, including zebrafish, mouse/rat, rabbit, cat, monkey, dog, miniature, and cellular models.

Overall, 1,099 papers were identified *via* searching. Forty-eight articles satisfied the inclusion and exclusion criteria and were reviewed for data collection. We categorized the animal models described in each study into eight groups, which will be discussed separately (data are summarized in Supplementary Table 1), including zebrafish, mice and rats, rabbits, monkeys, cats, dogs, pigs, and peripheral blood cell models (Figure 2).

### Zebrafish Model

It is well established that around 10%–15% of patients with MMD have a family history of MMD, while most cases are sporadic (4). The ring finger protein 213 (*RNF213*) gene is the first identified susceptibility gene in east Asian populations (5). Recently, the *RNF213* variant was also demonstrated to be related to the pathogenesis of various systemic vasculopathies within the pulmonary and cardiac vasculature (6, 7). Through the method of editing RNF213 gene, a series of experimental models were established in animal studies.

Zebrafish provides an ideal animal model for understanding the pathophysiology of human diseases (8). Zebrafish biology provides convenience for real-time imaging of developing pathologies, and researchers have ready access to all developmental stages of zebrafish. Given this, a wide range of zebrafish models has been generated for human diseases. Also, zebrafish is more economic than other vertebrate systems.

An RNF213 knockdown zebrafish model was generated by Liu et al., and the effects of RNF213 suppression on vasculature were investigated (9). Similar to the abnormal basal vasculature in MMD, knockdown zebrafish exhibited abnormal vascular development in the head but relatively normal vasculature in the trunk. The model specifically developed multiple aberrant vessels with irregular diameters originating from the inner optic circle and connecting to cranial vessels. Wen et al. (10) successfully generated *RNF213a* mutant zebrafish using the transcription activator-like effector nuclease (TALEN) technique. Abnormal vascular development in the cranial was observed. The *in vivo* model for MMD suggests that *RNF213* plays a crucial, selective role in intracranial angiogenesis. These zebrafish models would contribute to understanding the role of *RNF213a* in angiogenesis.

### Murine Model

Given the promising results demonstrated in zebrafish, attempts at creating a murine MMD model through *RNF213* were conducted. Murine models are the most commonly used laboratory animals, with a variety of methods employed to measure the development of MMD.

Homozygous knockout mice (*RNF213*−/−) were generated by deleting exon 32 of *RNF213* using the Cre–Lox system by Snonobe et al. (11). In contrast to the effect of *RNF213* knockdown in zebrafish, radiological evaluation through MRA of *RNF213−/−* mice did not reveal any gross malformations in the circle of Willis development or significant difference in thickness of vascular wall compared with wild-type mice. In a corroborating study with *RNF213* knock-in mice, Kanoke et al. (12) found no significant difference in the circle of Willis anatomy or MRA findings. Additional genes implicated in MMD include *ACTA2* and *NEO1*. The former encodes α-smooth muscle actin, while the latter encodes a transmembrane receptor or coreceptor for multiple ligands. Starosolski et al. (13) identified narrowing of the circle of Willis and abnormal straightening of large vessels in *ACTA2* mutant mice similar to the clinical pattern observed in MMD. *NEO1*-deficient mice have also recently been shown to have moyamoya-like vasculopathy, particularly the development of small, leaky, thin blood vessels within the cerebral cortex (14). The continued identification of susceptibility genes for MMD will provide novel genetic targets for the generation of animal models.

Except for gene mutations, inflammatory and infectious insults are postulated to be amplifying pathogenic mechanisms. Suzuki et al. (15) described the Wistar rats model that utilized intravenous or intrathecal injection of MDP and identified MMD-like pathological changes. Disruption of the internal elastic lamina and medial degeneration were present mainly in the intracranial ICA. However, this model failed to induce intimal thickening and stenosis. Yamada et al. (16) examined both the serum of MMD patients and the influence of inflammation in rats. In serum studies, elevated levels of several immunological factors were detected in MMD. In *in vivo* studies, *Propionibacterium acnes* was injected bilaterally around the carotid bifurcation in 7-day-old rats for 4 weeks. Histopathologically, moyamoya-like changes were demonstrated in intracranial ICAs. However, intimal thickening, medial thinning, and stenosis were not appreciated. While this model appears to recapitulate certain aspects of MMD, it fails to induce the progressive steno-occlusion and ischemia that are the hallmark consequence.

Given the underlying pathophysiological similarity between chronic vascular cognitive diseases and MMD, models of chronic hypoperfusion have been the mainstay for uncovering the brain’s response to ischemia and evaluating the effectiveness of surgical revascularization. For two-vessel occlusion of the bilateral common carotid arteries (CCAs) or ICAs, occlusion is usually achieved by ligation with 3-0 (rats) to 6-0 (mice) silk or nylon sutures of bilateral CCAs proximal to the bifurcation or distal ICAs (17–31). Single-vessel occlusion of the unilateral CCAs or ICAs is usually achieved by suture ligation of unilateral ICA (11, 12, 32–37). A series of ischemic consequences and/or treatments are described in these studies, such as growth factor application, EMS or burr hole surgery, and genetic therapies. However, the cerebral blood flow (CBF) decreases rapidly, produces an acute deficit, and is associated with high mortality up to 63% (25), especially in bilateral ligations of the CCAs. Thus, Mansour et al. developed a new modified CCA occlusion model, in which Sprague–Dawley rats underwent left CCA suture ligation and right CCA stenosis (26). A gradual CBF decline in the model results in a low mortality rate (2.3%) and cognitive impairment. With the discrepancy between the transient nature of these models and global progressive steno-occlusion MMD pathophysiology, the transient global ischemia (TGI) model was induced by occluding the CCAs for 5 min coupled with severe hypotension (38). Recently, Wang et al. (39) pioneered the implementation of a bilateral carotid artery stenotic mechanism compared to a bilateral carotid artery occlusion procedure in rats. From the microcoil perspective, Shibata et al. successfully induced CCH in *C57BL/6* mice through bilateral carotid artery stenosis using 0.18-mm microcoils (40). Besides, Roberts et al. demonstrated a novel internal carotid artery stenosis (ICAS) surgical technique to simulate MMS by placing microcoils on the proximal ICA in C57BL/6 mice (41). These results showed a significant narrowing of the distal ICA and anterior cerebral artery (ACA) in the circle of Willis and successfully mimicked the moyamoya-like vasculopathy seen in Suzuki stage I MMD as observed in humans. Importantly, it provides the first animal model created through surgical interventions specific for moyamoya vasculopathy. However, it is notable that the histological and pathological vascular profiles of these models do not fit those of MMD precisely. Despite the limitations of these animal models, they play a crucial role in the understanding of MMD.

The concept that one method alone may not be sufficient to promote or induce MMD has led to a discussion of combining different approaches. The work to unite the genetic and surgical pathways may be worthwhile to examine the effect of CCH in *RNF213* knockout mice. *RNF213* knockout mice that underwent CCA ligation demonstrated thinner intimal and medial layers compared with the control group (*P *< 0.05) (11). Ito et al. (42) implemented a transient middle cerebral artery occlusion tMCAO) model in *C57BL/6 RNF213* knockout mice and observed increased systemic angiogenesis because of the lack of an ideal MMD model that replicates both cerebral hypoperfusion along with angiogenesis at the base of the brain. A combined immunological–genetic model was reported by Kanoke et al., in which *RNF213−/−* mice were administered with adjuvants to determine whether an immunological reaction is an influential secondary insult in the development of MMD (43). However, the immunological stimulation failed to induce characteristic findings of MMD on gross inspection of the circle of Willis and by MRA of the cerebrovasculature. Morimoto et al. (44) performed bilateral CCA stenosis surgery using external microcoils on *RNF213* knockout and vascular endothelial cell-specific *RNF213* mutant transgenic mice; CBF restoration on day 28 and angiogenesis were significantly impaired in both groups compared with wild-type mice. Additionally, using the ICAS model pioneered by Roberts et al., the combination of such models with a transgenic mouse that expresses autoimmune disease could provide a model to understand both the disease progression and potential treatment modalities for moyamoya vasculopathy (41).

### Rabbit Model

Various early research studies indicated abnormal thrombogenesis and immune complex deposition within the vasculature of affected patients. Based on histopathological evidence confirming the presence of leukocytes and proliferating smooth muscle cells within the thickened intima of stenotic intracranial arteries from patients (45), MMD has been considered a variant of the vasculitic syndrome and/or autoimmune disease (46, 47). This discovery has also promoted the development of immunologically based animal models.

In light of inflammatory and immunological theory, Ezura et al. (48) induced a rabbit model by implementing a serum sickness vasculitis model combined with intracisternal administration of antibodies or antigens. The experiment included four groups: group I received twice intravenous injections of heterologous serum; group II received intracisternal administration of antibodies or antigens combined with a second injection of serum; group III was treated with a single intravenous injection of antigens simultaneously with intracisternal administration of antibodies; and group IV was set as control. Interestingly, any rabbit exposed to intracisternal heterologous serum exhibited transient features of cerebral arteritis with significant periarterial inflammatory cell infiltrate, while rabbits that were exposed to only intravenous heterologous serum did not develop any features of cerebral arteritis. Rao et al. (49) developed a similar rabbit model by chronic inoculation of horse serum for up to 1 year. The experiment included 21 Japanese rabbits in total. Horse serum was injected intravenously in one group, and the other group was injected locally near the sympathetic ganglia.

Histological changes involved intima stenosis of the lumen or even occlusion and disconnections of the inner layer. Moreover, hyperplasic smooth muscle cells extended inward through broken portions of the internal elastic lamina, and the proliferative vascular structure was also positive for IgG and IgM. Although the rabbit model appears to be a promising approach to recapitulate MMD, the requirement for long-term maintenance limits its ability to become widely adopted.

### Feline Model

For a cat, the carotid arterial system has extracranial and intracranial two components (50). The internal maxillary artery is the main supply vessel for the external part. The relatively large-sized intracranial ICA mainly received the blood supply from large-caliber anastomotic arteries, the cavernous sinus, and the primitive ICA. The circle of Willis differs from humans, and the anterior communicating artery was absent in cats. In the research, Kamijyo et al. described modifications in the filling pattern (50). Following unilateral short-term occlusion of the MCA with a clip, the main striate branches showed a good filling by contrast medium, as well as the circumferential branches over the hemispheric surfaces appeared. It was not designed for MMD, and no studies used the surgical method to induce the feline model.

Kamata et al. (51) attempted to build a feline model uniting an immune reaction and an arterial occlusion technique. Lactic acid–glycolic acid copolymer (LGA-50) is an immune-embolic material, and it is easy to be shaped. *N*-acetylmuramyl-L-alanyl-D-isoglutamine (MDP) is the main component of the bacterial cell wall, and it has the effect of inducing various biological effects or evoking an immune response (52, 53). Rod-shaped LGA-50 and MDP were injected into the unilateral CCA. Histologically, mild intimal thickening and duplication of the internal elastic lamina were observed, but these changes were minimal. Interestingly, the intimal changes of ICAs and their branches were found bilaterally, despite an injection of the immuno-embolic agent unilaterally. As more histological changes were presented in the prolonged experimental cats, the extent of the histological changes may relate to the duration and amount of immuno-embolic material administration. Moreover, the stenotic changes and development of moyamoya vessels were not expounded in the angiography of the feline model, despite a subset of the histological characteristics being replicated in the feline model.

### Monkey Model

As cerebrovascular system angiography showed, the terminal portion of the ICA and its branches of monkeys have the same morphology as the human carotid system. Terai et al. (54) also designed a new MDP method in the experimental research of monkeys. The MMD model was induced by intravascular interventional technique. Five monkeys received a repeated injection of the embolic materials in the right ICAs for all three groups. In the intravenous group, the monkeys underwent an intravenous repeated injection of MDP *via* a superficial vein of the forearm. In the control group, angiography was performed without any treatment. Reduplication of arteries and lamination of the internal elastic laminae were found both in embolic and intravenous groups. In the embolic group, these histological changes occurred bilaterally, and these were also observed both in the intracranial and extracranial arteries. However, neither stenosis of ICAs nor moyamoya vessels was observed.

### Canine Model

A dog is a major model species for cerebrovascular transformation research and shares similar anatomical features of brain vascular with humans (55). Suzuki et al. (56) divided 21 dogs into two groups and conducted an experiment that lasted for 1 month. The cat serum was injected intravenously in the first group, and the second group received a silicon capsule near the cervical sympathetic ganglion. Secretion of cat serum could stimulate the ganglion continuously until 1 mL of serum was consumed. Histological results showed that ICA changes correspond to human pathologic changes. The intimal thickening and internal elastic lamina duplication were observed in both groups, and immune complexes were observed in immunohistochemical staining of the intima and internal elastic lamina. Kasai et al. (57) carried out an experiment to demonstrate an immunological etiology of MMD in mongrel dogs. Their model induced serum sickness *via* injection of horse or cat serum. This operation would generate circulating immune complexes that could deposit within intracranial vasculature. Although histopathology failed to demonstrate the presence of immune complexes within these vessels, pathological arterial changes were identified in the terminal portion of the ICA. However, these changes are small enough to cause arterial stenosis. While this study asserted a likely role for inflammation in MMD, failure to identify intracranial immune complexes limited its pathogenic implications.

### Miniature Pig Model

The miniature pigs have similar brain structures to humans; they have been used in ischemic models associated with human stroke (58). For the cerebrovascular of miniature pig, it is easy to identify ICAs, ACAs, MCAs, PCAs, and anterior and posterior communicating arteries (59). Compared to the human vascular system, the MCA of miniature pig originates from the ICA in the cerebral hemisphere, one coursing laterally and another rostrally over the olfactory tract. The diameter remained constant with each other between the posterior communicating artery and ICA. It is suggested that the anterior circulation communicates well with the posterior circulation.

Nakamura et al. (37) set out a miniature pig model by internal carotid artery occlusion (ICAO). Fourteen pigs were used to investigate the chronological angiogenic changes and identify revascularization mechanisms. The results showed that a suitable environment and stimulus were required to induce angiogenesis in functional revascularization, for example, active vascular bed and ischemia. Yet, the animal model did not explicitly evaluate the cerebrovasculature, although the ICAO model of miniature pig was sufficient to discuss EMS-related revascularization.

### Cellular Model

More pieces of evidence suggest that MMD is probably a kind of hyperplastic vasculomyopathy (60), mainly an endometrial hyperplasia disease. From a cellular physiological standpoint, histopathological analysis indicates proliferation of both endothelial cells (ECs) and smooth muscle cells in the distal ICA (61–64). The first is the exploration of endothelial progenitor cells (EPCs). In 1997, EPCs were discovered in human peripheral blood for the first time (65). There is a general belief that EPCs originated from mesoderm cells during embryonic development (66); bone-marrow-derived progenitor cells could also differentiate into ECs lines (67). It is revealed that the abnormal number and function of endothelial progenitor cells (EPCs) were largely associated with the progression of MMD. Differentiation into ECs and remodeling of vascular ECM components are the ways for EPCs to play their functions after they pass through the endodermis (68, 69). For the number of EPCs, it was first found that the number of CD34+ cells in peripheral blood of MMD increased significantly in 2008 (70), and similar results were observed in other studies (71–73). However, the number of EPCs in the peripheral blood of MMD was often considered “controversial” because of two studies. One study showed a higher level of the EPCs using flow cytometry (71), while the other study revealed a lower level of EPCs employing colony-forming assays (74). First, MMD-specific induced pluripotent stem cells (iPSCs) carrying RNF213 R4810K were established by Hitomi et al. (75). The iPSCs were differentiated into ECs, and reduced angiogenic activities were detected for these ECs. It is suggested that the iPSC-derived vascular endothelial cells (iPSECs) is a promising *in vitro* model for MMD research. Kim et al. (76) also reported impaired angiogenic activity of EPCs in MMD, indicating that the phenotype of EPCs could be a disease-specific *in vitro* model. Furthermore, a study revealed disrupted mitochondrial morphology in the endothelial colony-forming cells (ECFCs) from the MMD, and the mitochondria seem to have shorter and more circular shapes and abnormal functions (77). In addition to the *in vitro* model described above, the only EPCs relevant *in vivo* experiment was conducted on rats (25). EPCs were obtained from MMD and were injected into the chronic cerebral hypoperfusion model. Nevertheless, less improvement in the cerebral perfusion, behavior, and amount of neovasculogenesis was found. Collectively, EPCs may be a potential target for *in vitro* model development.

The second referred to the crucial factors of smooth muscle progenitor cells (SMPCs) and smooth muscle cells (SMCs). The main histopathological finding in MMD was fibrocellular thickening of the intima, and the occlusion was essentially resulting from overproliferation of SMCs (78). Guo et al. (79) suggested that occlusive lesions and stenotic arteries of familial patients may be caused by smooth muscle tissue proliferation related to actin alpha 2 (*ACTA2*) mutations. The iPSC method was particularly conducive for diseases that the development of pathology has not elucidated. It is possible to explain the pathological mechanism after the establishment of the *in vitro* model of MMD. Kang et al. (80) purified SPCs from the peripheral blood of patients and demonstrated the development of irregularly arranged and thickened tubes in cell culture. The RNA was extracted from the cells, and differentially expressed genes (DEGs) were identified. These cells displayed specific DEGs compared with the control group. Recently, Tokairin et al. (81) characterized the VSMCs in MMD first in terms of the biological function and transcriptome profile. Both MMD and the control group displayed similar features of VSMCs, while ECs displayed distinct transcriptome profiles; the same EC features had also been validated previously (82). Therefore, SMCs may be another potential target for *in vitro* model development.

In addition, there was a close relationship between the EPC and SMC (83, 84). Overexpression of related factors in SMCs can help increase EPC adhesion and differentiation, migration, and proliferation (85, 86). However, according to the data in MMD research, the pathological changes are driven mainly by ECs, and VSMCs and specific environmental factors may play an important role (81). In 2017, C–C motif chemokine ligand 5 (CCL5) secreted by ECFCs was found to significantly augment the migration activities of SMCs toward ECFCs (87). Thus, EPCs contribute to neovascularization by paracrine effects. All in all, it is believed that these studies provide novel insight and cellular model for further research of MMD.

## Discussion

**Figure 3:**
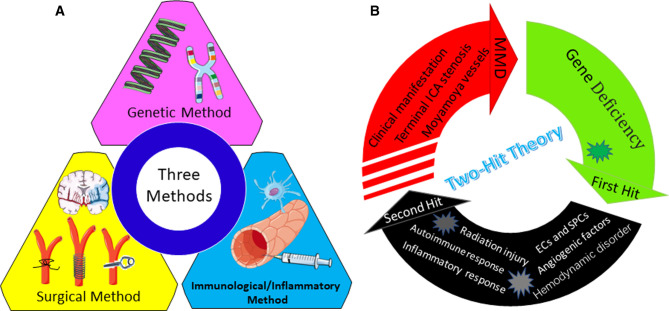
(**A**) Three approaches for generating moyamoya disease (MMD) animal models. (**B**) Possible two-hit theory underlying the development of MMD.

The complicated pathological features of narrow ICAs and the unknown nature of neovascularization indicate that the pathophysiology of MMD is a complex procedure. Advances made in defining pathophysiological aspects of MMD have set the foundation for the development of animal models to recapitulate the etiological mechanisms. As described previously, although a number of groups have attempted to induce MMD in zebrafish, mice or rat, rabbit, feline, canine, monkey, and miniature pig models, none were successful in replicating the full phenotype of MMD. Mice and rats were identified as the most frequently used animal models. The zebrafish model is a relatively new experimental model associated with gene mutations, given their unique external fertilization permitting direct observation of embryogenesis and their comparative ease of maintenance. Rabbit, cat, dog, and monkey models were mainly developed in the early stage of the MMD research in the last century. In the vascular anatomy aspect, the intracranial artery systems of all of the animal models are different from that of the humans except for the monkey model. An experimental model closer to the human cerebrovascular system may be further required in an investigation. As a primate, monkeys have a vascular system similar to humans. However, experimental models of primates are limited by cost, availability, and ethical considerations (88). Conversely, miniature pigs have advantages in these aspects compared with primates. Similar to the chronic ischemia of the murine models in mice or rats, the miniature pig models were mainly reported to understand the potential surgical treatment modalities for MMD at present. The preparation and procedures are relatively easier than normal pigs. In the review, three general approaches were mainly identified to develop various animal models, namely, genetic, immunological/inflammatory, and surgical approaches (78, 89) (Figure 3A).

From a genetic standpoint, genetic models were generally generated by various knockout, knockdown, or knock-in methods to study the impact of susceptibility genes on vasculogenesis, angiogenesis, and remodeling (9, 12, 42, 43, 90). A majority of genes have been described in the literature (91), such as the *RNF213* gene on chromosome 17q25, *ACTA2* gene on chromosome 10q23, *GUCY1A3* gene on chromosome 4q32, chromosome 3p, and 8q23, and so on. RNF213 polymorphism R4810K is the strongest susceptibility gene for MMD and exists in 0.5%–2.0% of the east Asian population (5, 9). It is reported that the R4810K variant is associated with ischemic MMD, whereas non-R4810K variants are related to hemorrhagic MMD, especially A4399T (92). Notably, gene knockout and/or knock-in model of the susceptibility gene construct did not sufficiently develop MMD, indicating that additional environmental factors may play a critical role; “two-hit theory” was considered for the development of MMD (93). A study revealed that there were no obvious abnormalities both in imaging and histopathology of the vascular in *RNF213*−/− mice. However, significantly thinner of the intima and medial layers were observed in *RNF213*−/− 14 days after CCA ligation, no significant difference was observed on 7, 21, and 28 days after CCA ligation (11).

At the time of onset, “first hit” may be characterized by RNF213 deficiency. Next, the “second hit” appeared in the form of autoimmune response, infection or inflammation, radiation, mitochondrial dysfunction, oxidative stress, hemodynamic disorder, and so on. As the *RNF213* deficiency vascular wall is more vulnerable to the “second hit,” progressive stenosis of ICAs and abnormality of the cerebral vascular network can develop and ultimately lead to MMD. In the future, the “multiple-hit model” appears to be widely accepted for recapitulating the complexity of MMD with further research, involving the interaction of genetic and environmental factors, crosstalk between different tissues and cells, and the influence of angiogenic factors and pro-inflammatory molecules (Figure 3B).

From an immunological standpoint, immunological models attempt to reproduce the morphological changes observed in the intracranial vasculature of patients with MMD (16, 51). The frequency of 1.7–4.7% in adults and 0.54–1.5% in infants with an inflammatory disease concurrent with MMD (94). A high prevalence of autoimmune disorders and elevated levels of autoimmune antibodies in blood revealed that the MMD could have some relationship with autoimmune diseases. The immune response caused by autoimmune thyroid disorders (95), diabetes syndrome (96), or systemic lupus erythematosus (SLE) (97) may participate in the pathological process of MMD. Kim et al. (98) reported the elevated levels of thyroid autoantibodies in MMD patients, indicating that immune disorders are involved in the progression of MMD. Furthermore, anti-inflammatory cytokines and proinflammatory cytokines are two major pathways in the inflammatory response for the development of MMD. It is revealed that the proinflammatory cytokine pathway is activating RNF213 functions distinct from the anti-inflammatory cytokine pathway (99). In addition, researchers have identified chronic inflammation aroused by immune responses as a major part of the MMD pathological process (100). However, the molecular mechanisms of the inflammatory/immune response in MMD still have not been fully clarified.

From a surgical standpoint, surgical models utilize mechanical occlusion or narrowing of extracranial and/or intracranial vasculature to replicate stenosis and accompanying cerebral ischemia (22, 28). Direct, indirect, and combined surgical revascularization is still the first choice for patients. A number of techniques have been developed to recreate chronic cerebral hypoperfusion animal models through occlusion of the CCAs and ICAs (101–104). Artery occlusion remains the most common method of simulating the ischemic microenvironment of moyamoya in mice, rats, and pigs. The clinical treatment modalities explore with the assistance of these ischemic models. The effect of indirect revascularization, especially EMS, was evaluated in detail by two-vessel occlusion (17, 18, 20, 21, 24, 28, 29), single-vessel occlusion (32, 35, 36), and microcoil (105) methods of ischemic models. Angiogenetic microRNA, gene therapy, myoblast implantation, and bone marrow stromal cells are confirmed to have the potential as a new therapeutic strategy in the chronically hypoperfused brain. Moreover, the underlying mechanism of burr hole surgery was also evaluated by two-vessel occlusion (27) and tMCAO (106, 107) methods of ischemic models. This treatment can provide feasible treatment options for MMD patients. Despite the benefits of surgical models, several limitations exist, such as a sudden reduction in challenges to achieving satisfactory reproducibility.

While these models reproduce the disparate aspects of MMD independently, no model exists that satisfactorily combines genetic predisposition, immunologically mediated vasculopathy, and chronic ischemia. Recently, EPCs and SPCs have attracted much attention in the study of the pathogenesis of MMD because of the difficulty in obtaining specimens and a lack of animal models (108). MMD is a unique disease that involves the proliferation, migration, differentiation of different vascular cells, and the maintenance of the vascular wall. Various types of cells, but not limited EPCs and SPCs, may play a complex role in the MMD process. Different cell types may be recruited during the aberrant angiogenesis process, such as SMCs (80), SMPCs (70), circulating ECs (109), and immune cells (45), eventually leading to stenosis and abnormal collateral formation. Besides, *RNF213* may serve as a downstream medium of the IFN-β signaling pathway and function as a common responder of the PI3 kinase-AKT signaling pathway in ECs (7, 110). At last, the existing controversies about the EPCs in different studies were mainly caused by different classification methods and isolation/cultivation strategies. Thus, the inconsistent results lack comparability. All in all, additional research in the cellular model would complement other animal models and greatly enhance the limited understanding of the pathology underlying MMD.

## Conclusion

To date, a variety of effective experimental models have been trailed by different animal species and approaches in an attempt to understand both the disease progression and potential treatment modalities for MMD. Animal models described in each study categorize as genetic mechanisms, immune-mediated arterial injury, and mechanical occlusion or stenosis within the cerebral vasculature based on the methods used to recapitulate MMD pathogenesis. Each animal model has its unique advantages and disadvantages. Unfortunately, none were successful in replicating the full phenotype of MMD. Mice and rat models are the most common animal models in MMD research. In the future, cellular models may provide a new perspective for understanding the etiology and pathology of MMD.
