# Supplementary material for: Experimental Animal Models for Moyamoya Disease: A Species-Oriented Scoping Review
Source: Front Surg. 2022 Jul 1;9:929871. doi: 10.3389/fsurg.2022.929871 (PMC9283787; doi:10.3389/fsurg.2022.929871)
Supplement: Supplementary file 1 [file Table_1_v1.docx]

| **species** | **Num.** | **Authors & year** | **models** | **Description of methods** | **conclusions** |
| --- | --- | --- | --- | --- | --- |
| Zebrafish | 1 | Liu et al., 2011^[4]^ | RNF213 knockdown | Knockdown of *RNF213-α* and *RNF213-β* expression was achieved by injection of a specific morpholino into 1- to 8-cell stage embryos | Large trunk arteries formed almost normally but these vessels were of an irregular diameter and showed aberrant sprouting, *RNF213* morphants sprouted abnormal vessels from the optic vessels at 60-72 hpf |
|  | 2 | Wen et al., 2016^[5]^ | RNF213 knockout model | generated rnf213a mutant zebrafish using transcription activator-like effector nuclease technique | Abnormal angiogenesis in intersegmental vessels and cranial secondary vessels. Endothelial cells exhibited the defects in morphogenesis and formation of vascular tubes. Circulatory disorder in the trunk and head, lower erythrocyte velocity in dorsal aorta |
| Rats | 3 | Suzuki et al., 1987^[6]^ | Inflammatory | intravenous or intrathecal injection of MDP | Disruption of internal elastic lamina, medial necrosis, predominantly w/in terminal ICA |
|  | 4 | Yamada et al., 1997^[7]^ | Infectious | Injection of rats with P. acnes in area surrounding bilat carotid bifurcations | Moyamoya-like changes of the ICA arteries were histopathologically demonstrated, P. acnes and immunological factors might play a role in the pathogenesis |
|  | 5 | Kusaka, N. et al.,2005^[8]^ | Two-vessel occlusion | Bilateral CCAs ligated with 3-0 silk sutures combined EMS procedures | Administration of phVEGF may be an effective therapy in patients with chronic cerebral hypoperfusion |
|  | 6 | Matsuda, T. et al.,2005 ^[9]^ | Two-vessel occlusion | Bilateral ICA ligated with 3-0 silk sutures combined EMS procedures | Neovascularization for therapeutic collateral circulation may be achieved using a transcriptional regulatory strategy |
|  | 7 | [Ohtaki](https://pubmed.ncbi.nlm.nih.gov/?term=Ohtaki+H&cauthor_id=16671472) et al.,2006^[10]^ | Two-vessel occlusion | The CCAs were doubly ligated with 3-0 silk suture and the arteries cut between the sutures | Chronic hypoperfusion results in mechanisms to compensate for insufficiency in blood flow including vasodilation, VEGF expression, and neovascularization in the ischemic region |
|  | 8 | Kim, H.S., et al.,2008 ^[11]^ | Two-vessel occlusion | Bilateral CCAs ligated with 3-0 silk sutures | The injection of bone marrow stromal cells resulted in accelerated angiogenesis in the temporal muscle |
|  | 9 | Ohmori, Y. et al.,2011 ^[12]^ | Two-vessel occlusion | Bilateral ICA occlusion ligated with 4-0 silk sutures combined encephalogaleosynangiosis | Combined use of G-CSF and indirect bypass surgery induces an increase in rCBF and angiogenesis under conditions of cerebral chronic hypoperfusion |
|  | 10 | Gong et al., 2013^[13]^ | Two-vessel occlusion | both ICA were exposed and double knots were made for permanent ligation with 4-0 silk sutures | brain vasculature in young rats has plasticity to external insult caused by cerebral hypoperfusion. |
|  | 11 | Nam et al.,2015^[14]^ | tMCAO | A 3-0 monofilament nylon suture with a rounded tip was introduced through the left ECA and was advanced to the ICA until slight resistance was felt. A burr hole was made seven days after the ischemic injury | Bone marrow-derived stem cells through burr holes seem to play an important role for the therapeutic effect of the multiple cranial burr hole surgery in MMD. |
|  | 12 | Su et al.,2015^[15]^ | Two-vessel occlusion | The CCAs were doubly ligated with 3-0 silk suture | WIN55,212-2 and [URB597](https://www.sciencedirect.com/topics/neuroscience/urb597) protect neurons against chronic ischemic insults |
|  | 13 | Sato-Maeda et al.,2017^[16]^ | transient global ischemic | Both of the [common carotid arteries](https://www.sciencedirect.com/topics/medicine-and-dentistry/common-carotid-artery) were occluded using [aneurysm clips](https://www.sciencedirect.com/topics/nursing-and-health-professions/aneurysm-clip) for 5 minutes while maintaining blood pressure at between 25–30 mmHg | *Rnf213* was upregulated in the vulnerable neurons of the [hippocampus CA1](https://www.sciencedirect.com/topics/medicine-and-dentistry/sommers-sector) subregion and ischemic cortex after [tGCI](https://www.sciencedirect.com/topics/medicine-and-dentistry/brain-ischemia). |
|  | 14 | Hiramatsu, M., et al. ,2017 ^[17]^ | Two-vessel occlusion | Bilateral common carotid arteries ligation combined EMS procedures | Combined gene therapy (VEGF plus apelin) during EMS in a chronic cerebral hypoperfusion model can enhance angiogenesis |
|  | 15 | Choi et al.,2018^[18]^ | Two-vessel occlusion | The CCA were double-ligated with 4-0 silk combined ECFCs injection | The fine-tuning of an animal model would be meaningful, abnormalities of ECFCs in terms of neovascularization and neurogenesis might lead to MMD. |
|  | 16 | [Mansour](https://pubmed.ncbi.nlm.nih.gov/?term=Mansour+A&cauthor_id=30192196) et al.,2018^[19]^ | Two-vessel occlusion | The left CCA was permanently occluded using a 6-0 silk suture, and the right CCA was gently banded with a 6-0 silk suture. | The modified CCA occlusion model induced one-sided occlusion and contralateral side stenosis in the early stage, the stenosis side was spontaneously occluded resulting in 17bilateral occlusion, a straightforward method of pr18oducing a CCH model in rats |
|  | 17 | Nishihiro, S., et al.,2019 ^[20]^ | Single-vessel occlusion | CCAs were ligated with 3–0 silk sutures combined EMS procedures | High19-mobility group box-1 with EMS promoted brain angiog20enesis in a VEGF-dependent manner, resulting in cerebral blood flow improvement |
|  | 18 | Park, G.H et al.,2019^[21]^ | Two-vessal occlusion | Bilateral ICA ligated using 4–0 silk sutures combined cranial burr hole with small dural cracks | Cranial burr hole and erythropoietin (EPO) generate effective revascularization |
|  | 19 | Chen, C., et al.,2020^[22]^ | two-vessel occlusion | Bilateral common carotid artery ligated with two 3-0 silk threads | Increasing miR-126-5p expression in the temporal muscle can promote EC proliferation and angiogenesis through the PI3K/Akt pathway |
|  | 20 | Li, W. et al.,2021 ^[23]^ | Two-vessel occlusion | Bilateral of CCA ligated with two 4-0 silk threads combined EMS procedures | The degree of endothelial cell (EC) proliferation and CBP improvement peaked at 4 weeks after EMS on the ipsilateral side, whereas the cognitive improvement peaked in the fifth week |
|  | 21 | Wang et al.,2021^[24]^ | Two-vessel occlusion | The CCAs were doubly ligated with 4-0 silk suture combined EMS | Augmentation of endogenous cannabinoid signaling but not EMS protects against CCH-induced neurodegeneration and preserves spatial learning and memory. |
| Mice | 22 | Hecht et al.,2011^[25]^ | Single-vessel occlusion | Right-sided ICA was ligated with 8/0 Nylon combined EMS | The implantation of genetically modified myoblasts into an EMS represents a promising novel strategy for the local delivery of gene products to the muscle/brain interface and the cerebral cortex |
|  | 23 | Sonobe et al.,2014^[26]^ | Single-vessel occlusion combined *RNF213*-deficien | *RNF213*-deficient mice were generated by deleting exon 32 of *RNF213.* The left CCA was ligated proximal to the bifurcation using 5-0 silk. | mice lacking the RNF213 gene did not spontaneously develop MMD |
|  | 24 | Sonobe et al., 2014^[27]^ | CCA ligation combined RNF213 knockout | Generated knockout mice by deletion of exon 32 of RNF213 gene, the left CCA was ligated just proximal to the bifurcation using 5-0 silk. | The increased vascular expression of MMP-9 and subsequent vascular wall thinning in RNF213-/- mice were observed |
|  | 25 | Kobayashi et al., 2015^[28]^ | Mouse RNF213-R4757K overexpression model | generated mice that overexpress R4757K variant of RNF213 | Angiogenesis was not induced in mice overexpressing R4757K w/in endothelial cells after hypoxia whereas it was present in other mice |
|  | 26 | Starosolski et al., 2015^[29]^ | ACTA2 knockout | Generated heterozygous and knock-out mice | Narrowing & straightening of large vessels w/in circle of Willis identified by a novel imaging approach |
|  | 27 | Kanoke et al.,2015^[30]^ | Single-vessel occlusion combined RNF213-R4828K knock-in | RNF*213* R4828K knock-in mice were generated , and the [CCA](https://www.sciencedirect.com/topics/neuroscience/common-carotid-artery) was ligated just proximal to the bifurcation with 6–0 silk | No cerebrovascular defect visible. No difference in thickness of intracranial vasculature or in vascular remodeling following CCA ligation. Multiple secondary insults may also contribute to the onset of MMD |
|  | 28 | Ito et al.,2015^[31]^ | tMCAO  combined RNF213 knockout | *RNF213* knockout mice were generated by deleting exon 32 of *RNF213* with the Cre/loxP system, and transient [middle cerebral artery](https://www.sciencedirect.com/topics/neuroscience/middle-cerebral-artery) occlusion (tMCAO) were conducted by the intraluminal suture technique | [Angiogenesis](https://www.sciencedirect.com/topics/neuroscience/angiogenesis) was enhanced in mice lacking *RNF213* after chronic hind-limb ischemia, which suggested the potential role of the *RNF213* gene abnormality in the development of abnormal vascular networks in chronic ischemia. |
|  | 29 | Hecht, N., et al.,2015 ^[32]^ | Single-vessel occlusion | Right-sided ICA ligated with an 8/0 silk suture combined EMS procedures | Myoblast-mediated VEGF supplementation at the target site of an EMS improved surgical revascularization results and provide protection from ischemic stroke |
|  | 30 | Sato-Maeda et al.,2016^[33]^ | tMACO | The [ICA](https://www.sciencedirect.com/topics/neuroscience/internal-carotid-artery) and CCA were temporarily closed using a vascular clip, and a silicon-coated 6-0 nylon monofilament was then introduced into the arteriotomy hole in the ECA and advanced into the CCA bifurcation. After removing the vascular clip and cutting the ECA, the intraluminal suture was inserted into the ICA approximately 9 mm from the CCA bifurcation until mild resistance was felt. | The Rnf213 gene was up-regulated in the ischemic brain as early as 6 h after [tMCAO](https://www.sciencedirect.com/topics/neuroscience/middle-cerebral-artery). |
|  | 31 | Kanoke et al., 2016^[34]^ | Immunology combined RNF213 knockout | Generated knockout mice by deletion of exon 32 of RNF213 gene.  MDP-Lys (L18), CFA, and PC61 were used as immunological adjuvants and were injected | significant reduction in ratio of regulatory T cells after administration of immunological adjuvant (MDP) but did not demonstrate characteristic findings of MMD |
|  | 32 | Morimoto et al.,2018^[35]^ | Microcoils  Combined RNF213 knockout | Microcoils with an internal diameter of 0.18 mm were applied to the bilateral common carotid arteries among *Rnf213* knockout mice, endothelial cell-specific p.R4757K transgenic mice and wild type mice | *RNF213* plays an important role in CBF maintenance under ischemic conditions by affecting angiogenesis and arteriogenesis. A chronic hypoperfusion model such as BCAS could be a promising model for investigating the role of *RNF213* in steno-occlusive diseases, including MMD. |
|  | 33 | Roberts et al.,2018^[36]^ | Microcoils | The occipital artery was permanently ligated with fully exposed CCA, ECA and ICA, micro-coils with an internal diameter of 0.16 millimeter were placed on the proximal ICA | A new surgical technique termed internal carotid artery stenosis (ICAS) were demonstrated to mimic MMS using micro-coils on the proximal ICA |
|  | 34 | Hayashi, T., et al., 2020^[37]^ | Microcoils | Bilateral common carotid artery stenosis (BCAS) with 0.16-mm-diameter microcoils combined EMS procedures | PDGFRα signal may play an important role in developing spontaneous angiogenesis between the temporal muscle and neocortex after EMS in moyamoya disease |
|  | 35 | [Marushima](https://pubmed.ncbi.nlm.nih.gov/?term=Marushima+A&cauthor_id=30621518) et al.,2020^[38]^ | Single-vessel occlusion | The right-sided ICA was permanently ligated with an 8/0 silk suture combined with EMS and myoblast implantation | EMS surgery together with myoblast-mediated co-delivery of VEGF/PDGF-BB may have the potential to serve as a novel treatment strategy for augmentation of collateral flow in the chronically hypoperfused brain. |
|  | 36 | Ren et al., 2021^[39]^ | NEO1 knockout model | [Neogenin](https://www.sciencedirect.com/topics/medicine-and-dentistry/neogenin) mutant mice were generated by Ozgene | Astrocytic Neo1-loss resulted in an increase of small blood vessels selectively in the cortex, with leaky blood-brain barrier (BBB), thin arteries, and accelerated [hyperplasia](https://www.sciencedirect.com/topics/medicine-and-dentistry/hyperplasia) in veins and capillaries |
| Rabbits | 37 | Ezura et al., 1992^[40]^ | Serum sickness vasculitis model | Presensitization via intravenous injection of horse serum followed by intracisternal injection of horse serum and anti–horse serum antibodies | cerebral arteries rarely develop arteritis in a serum sickness model alone and additional intracisternal administration of antibodies or antigens may required |
|  | 38 | Rao et al., 2003^[41]^ | Serum sickness vasculitis model | injections of horse serum either intravenously or locally in the area of the sympathetic ganglia | Intimal hyperplasia, elastic degeneration, deposition of immunoglobulins with terminal ICA; extension of hyperplastic smooth muscle cells through internal elastic disruptions resulting in luminal stenosis |
| Cats | 39 | Kamata et al., 2003^[42]^ | Inflammatory | LGA-50, MDP and immuno-embolic material, were injected into cats unilaterally via the common carotid artery. | Histological changes of duplication of the internal elastic lamina could be seen mainly in the terminal portion of the ICA, no angiographic changes were seen |
| Monkeys | 40 | Terai et al., 2003^[43]^ | Inflammatory | Immunoembolic injection of MDP in a rodshaped lactic acid/glycolic acid (50:50) copolymer following sensitization with intravenous injection of MDP | reduplication and lamination of the internal elastic laminae, occurred in the intracranial arteries on the embolization side, the contralateral intracranial and even extracranial arteries. |
| Dog | 41 | Suzuti et al.,1976^[44]^ | Serum sickness vasculitis model | One group received the intravenous injection of cat serum, while the other group placed a silicon capsule containing 1 ml cat serum near the cervical sympathetic ganglion. | Thickening of the intima and duplication of the internal elastic lamina, and immune complex in tunica intima and internal elastic lamina |
|  | 42 | Kasai et al., 1982^[45]^ | Serum sickness vasculitis model | multiple intravenous or subcutaneous injections of horse or cat serum | Intimal thickening, elastic lamina folding/ tortuosity, medial thinning/necrosis; localization of changes to terminal ICA, consistent w/ MMD. Failed to identify immune complexes in vessel wall. |
| Miniature pig | 43 | Nakamura M et al, 2009^[46]^ | Single-vessel occlusion | Electrocautery ligation of unilateral internal carotid artery occlusion combined EMS procedures | Functional revascularization requires a suitable environment and stimulus to induce vascular expansion |
| Cellular model | 44 | Kim et al.,2010^[47]^ | endothelial progenitor cells | Peripheral blood mononuclear cells (PBMNCs) were isolated and cultured in endothelial cell growth medium | the circulating EPCs reveals decreased level and defective function |
|  | 45 | [Hitomi](https://pubmed.ncbi.nlm.nih.gov/?term=Hitomi+T&cauthor_id=23850618) et al.,2013^[48]^ | Induced pluripotent stem cells (iPSCs) - derived vascular endothelial cells | Dermal fibroblasts were isolated from arms and cultured, then induced the differentiation of iPSCs into vascular endothelial cells | RNF213 R4810K reduced angiogenic activities of iPSECs from patients with MMD, suggesting that it is a promising *in vitro* model for MMD. |
|  | 46 | Kang et al.,2014^[49]^ | smooth-muscle progenitor cells | Cultured and isolated SPCs from the peripheral blood of patients with MMD | SPCs could be established from the peripheral blood of patients with MMD. These cells showed specific DEGs compared with healthy control volunteers |
|  | 47 | Hamauchi, S., et al.,2016^[50]^ | endothelial cells | Endothelial differentiation was performed from the peripheral blood of three patients with MMD carrying the variant RNF213 R4810K using induced pluripotent stem cell (iPSC)-derived ECs | Angiogenesis was significantly impaired in MMD, endothelial proliferation was not significant between control- and MMD-derived cells. pathway analysis illustrated that extracellular matrix (ECM) receptor-related genes, were significantly downregulated, Proteomic analysis revealed that cytoskeleton-related proteins were downregulated and splicing regulation-related proteins were upregulated |
|  | 48 | Tokairin, K., et al.,2020^[51]^ | vascular smooth muscle cells | VSMCs were differentiated from neural crest stem cells (NCSCs) using peripheral blood mononuclear cell using induced pluripotent stem cell (iPSC)-technology | similar biological function and transcriptome profile of iPSC-derived VMSCs in MMD patients and HC subjects, displaying distinct transcriptome in the ECs |
